# The temporal change of heat exposure and adaptation capacity in Chinese adults from 1994 to 2023

**DOI:** 10.3389/fpubh.2024.1492523

**Published:** 2025-01-28

**Authors:** Xiaohui Ji, Haomin Tan, Shaoli Huang, Zhongguo Huang, Jianxiong Hu, Guanhao He, Fengrui Jing, Ziqiang Lin, Mengen Guo, Tao Liu, Wenjun Ma

**Affiliations:** ^1^Department of Public Health and Preventive Medicine, School of Medicine, Jinan University, Guangzhou, China; ^2^Key Laboratory of Viral Pathogenesis and Infection Prevention and Control, Ministry of Education, Jinan University, Guangzhou, China

**Keywords:** ambient temperature, adaptation capacity, global warming, health risk, the temporal change

## Abstract

**Background:**

Studies have found decreased heat effect and increased minimum mortality temperature (MMT) during the past decades. However, it is unclear whether heat exposure or temperature adaptation play an important role in this change.

**Methods:**

This is a cross-sectional study. Data were collected from 3,094 respondents aged 31–64 years old based on online questionnaire. The Cochran-Armitage test for trend and Cochran–Mantel–Haenszel (CMH) test were used for the difference between three decades. The Chi square test was employed to compare the difference between different demographic subgroups during 2014–2023. Multivariate logistic regression model was used to analyze the risk factors of air conditioner ownership.

**Results:**

Most respondents (94.6%) thought ambient temperature had been increasing, and 57.0% people thought climate change impacted their health. Long duration outdoors work (≥4 h) decreased from 36.01, 30.93 to 24.53% (Z = −9.80, *p* < 0.01) and bicycling/walking decreased from 62.3, 27.9, to 9.7% (CMH value = 156.40, *p* < 0.01) significantly during the last three decades. Temperature adaptation capacity increased with air conditioner ownership rates increasing from 25.40, 57.63 to 81.51% at home (Z = −44.35, *p* < 0.01) and from 22.24, 57.47 to 80.51% in the office/school (Z = −45.95, *p* < 0.01), and the older adult, women, people with low income, outdoor work, low education, and people from northern China had lower air conditioner ownership rates. The frequency of air conditioner usage when felt hot also escalated significantly both at home (from 42.6%, 54.9, to 63.4%, CMH value = 156.40, *p* < 0.0001) and in the office/school (from 61.8, 63.1 to 72.7%, CMH value = 65.29, *p* < 0.0001) during the same periods.

**Conclusion:**

Our study found that most people perceived climate change and changed behaviors to adapt to heat. Heat exposure significantly decreased and temperature adaptation capacity significantly increased during the last decades. The findings implied that heat-related health risk and burden driven by global warming may not increase in the future.

## Background

In the sixth climate change assessment report in 2021, the Intergovernmental Panel on Climate Change (IPCC) reported that global climate would continue to warm, and the frequency and intensity of extremely hot events might continuously increase in the future (1). Many studies found that non-optimum temperature, namely the temperature above or below the temperature with minimum mortality risk (2), could increase human health risk and disease burden. For example, a national study in China found that non-optimum temperature could lead to 1.02 years life loss per death (3–5). However, the adverse effect of non-optimum temperature might be reduced through temperature adaptation over time (6). The IPCC defines temperature adaptation as the natural adjustments made by human systems to adapt to new or constantly changing environments (7). Temperature adaptation includes physiological, technological and behavioral adaptation. Physiological adaptation is limited. For instance, when extremely high temperature exceeds physiological adaptation capacity, people need technical or behavioral adaptation to reduce health risk (8).

With economic development, technological advancement and health care service improvement, population adaptation capacity increased (2, 9). For instance, although many studies have reported that increased temperature has escalated mortality risk, heat effect has significantly declined over the past decades (2, 10–12) and the minimum mortality temperature (MMT), namely temperature at which the lowest mortality occurs increased (13–18). For instance, Elisaveta et al. found that in the USA, the health risk of high temperature decreased gradually from 1970 to 2006 (19). Another study in Japan reported that MMT increased by about 5.5°C from 1972 to 2012 (16). However, it is unclear what drives the decrease. One of the possible hypotheses was the improvement of adaptation capacity (2, 20–22), but there was lack of empirical research, especially in developing countries. China, a large country with many climate zones, has seriously been affected by climate change. In recent decades, rapid socio-economic and technological development may improve adaptation capacity of Chinese population (23). However, there was no study on the temporal change of health adaptation capacity in China. Therefore, exploring the role of the temporal trend of adaptation capacity on the declined heat effect is very necessary, which can better inform climate change policy development and implementation.

In the present study, we conducted an online survey to investigate the current knowledge, attitude and practice (KAP) on climate change and the temporal change of heat exposure and adaptation capacity among Chinese adults from 1994 to 2023. Our findings will provide new evidence on the importance of taking effective climate change adaptation action in the context of global warming.

## Materials and methods

### Study design and sample

This cross-sectional study was conducted using an online questionnaire survey to collect KAP on climate change, the changing trend of heat exposure and adaptation capacity among Chinese adults from 1994 to 2023. Data were collected online via self-reporting based on Questionnaire Star. Given the high internet usage in China, a survey Quick Response (QR) code was distributed to respondents via WeChat Moments and Friend groups. Participants retrospectively filled in the questionnaire by scanning the QR code.

### Questionnaire development

The self-reported questionnaire was developed by our research team. In order to ensure the quality of the questionnaire, we conducted an expert consultation meeting and a pilot study to improve the questionnaire before formal investigation. On the first page of the questionnaire, participants were clearly informed about the background and objectives of the study. Each participant agreed to participate in the study and signed an informed consent form. In order to reduce recall bias in the retrospective self-reporting, we reminded the respondents that three periods from 1994–2003, 2004–2013, to 2014–2023 were corresponded to three top leaders in China.

The structured questionnaire included 4 parts: socio-demographic characteristics, knowledge and practice on climate change during 2019–2023, heat exposure and adaptation capacity during 1994–2003, 2004–2013 and 2014–2023. Specifically, the socio-demographic characteristics included age, sex, education, income, family address, and the number of chronic diseases. The knowledge on climate change is about the health risk of climate change, and the frequency of obtaining heat information (24, 25). The adaptation practice means the behaviors to prevent diseases associated with high temperature (26, 27). The heat exposure included hours working outdoors in summer and main types of daily transportation from 1994 to 2023. The temperature adaptation capacity included the ownership rate of air conditioner and the usage frequency of air conditioners in summer at home or in the office/school.

### Statistical analysis

Categorical data were described as frequencies and proportions. A Bar Chart was used to show knowledge and practice on climate change. We used Stacked Bar Chart to describe the percentage changes in temperature adaptation capacity from 1994 to 2023. The Cochran-Armitage test for trend was used to compare rates over three decades. The Cochran–Mantel–Haenszel test was employed to test the difference of ordered variable between the three decades. The Chi square test was used to compare the difference between different demographic subgroups during 2014–2023. We also used multivariate logistic regression model to analyze risk factors of air conditioner ownership rate and usage frequency during 2014–2023. Statistical analysis was conducted using R 4.2.2 software. Two-sided *p* < 0.05 was statistically significant.

## Results

### The characteristics of the study sample

A total of 3,094 respondents was included in the study with 1,545 respondents aged 31–40 years old and 1,549 respondents aged 41–64 years old. Table 1 presents the characteristics of the participants in this study. Females (57.5%) were more than males, and 76.9% participants worked indoors. Most respondents (85.2%) were above undergraduate. The monthly income of 8.7% participants was more than 20,000 yuan. Most participants (63.8%) did not have any chronic diseases.

**Table 1:** Basic characteristics of the participants in this study.

Subgroups	Total	31–40 years old	41–64 years old
	*N*(%)	*N*(%)	*N*(%)
Total	3,094(100.0)	1,545(49.9)	1,549(50.1)
Sex			
Male	1,314(42.5)	659(42.7)	655(42.3)
Female	1,780(57.5)	886(57.4)	894(57.7)
Occupation			
Main indoor	2,378(76.9)	1,201(77.7)	1,177(76.0)
Half indoor and half outdoor	514(16.6)	230(14.9)	284(18.3)
Main outdoor	202(6.5)	114(7.4)	88(5.7)
Education			
Graduate or above	511(16.5)	338(21.9)	173(11.2)
Diploma/undergraduate	2,126(68.7)	1,036(67.1)	1,090(70.4)
High school	337(10.9)	131(8.5)	206(13.3)
Junior high school	99(3.2)	33(2.1)	66(4.3)
Primary school and below	21(0.7)	7(0.5)	14(0.9)
Income (yuan)			
>20,000	269(8.7)	136(8.8)	133(8.6)
10,001–20,000	605(19.6)	315(20.4)	290(18.7)
5,001–10,000	1,191(38.5)	604(39.1)	587(37.9)
2001–5,000	906(29.3)	442(28.6)	464(30.0)
<2000	123(4.0)	48(3.1)	75(4.8)
Number of chronic diseases			
0	1,973(63.8)	1,147(74.2)	826(53.3)
1	711(23.0)	280(18.1)	431(27.8)
2	286(9.2)	95(6.2)	191(12.3)
≧3	124(4.0)	23(1.5)	101(6.5)

### The knowledge and practice on climate change and health

**Figure 1:**
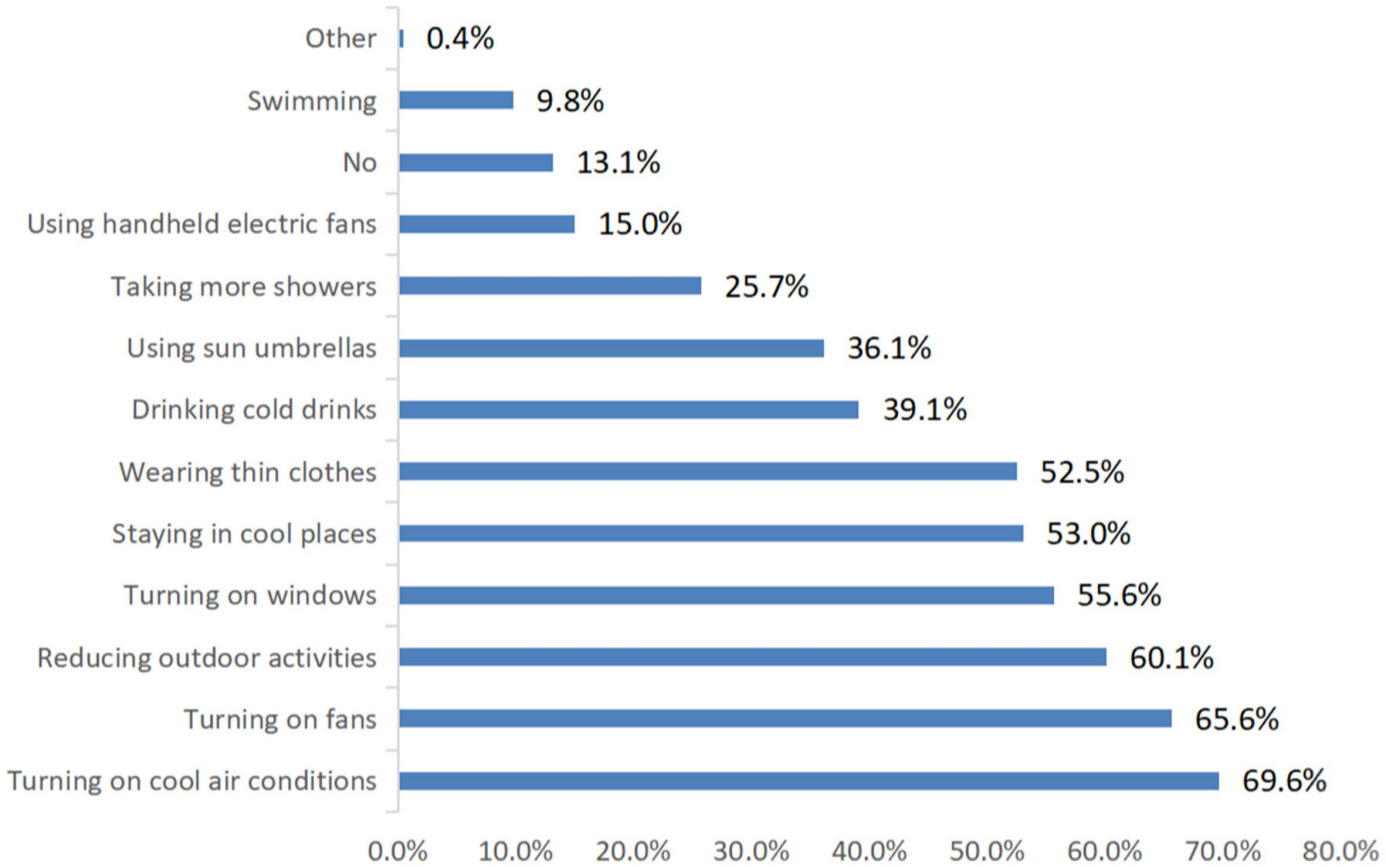
The practices of adaptation to climate change among the respondents in China in the past 5 years.

During 2019–2023, 60.2% participants knew the true meaning of climate change. There were 48.5 and 44.5% participants paying attention to heat information more than 5 days and 1–4 days a week, respectively, and 94.6% participants perceived increased temperature compared with their childhood, 57.0% participants thought that the health impact of climate change was significant. In terms of adaptation practice (Figure 1), using air conditioners and fans, reducing outdoor activities, opening windows, staying in cool or air-conditioned areas were top 5 adaptation practices.

### The temporal change of heat exposure during the last three decades

**Figure 2:**
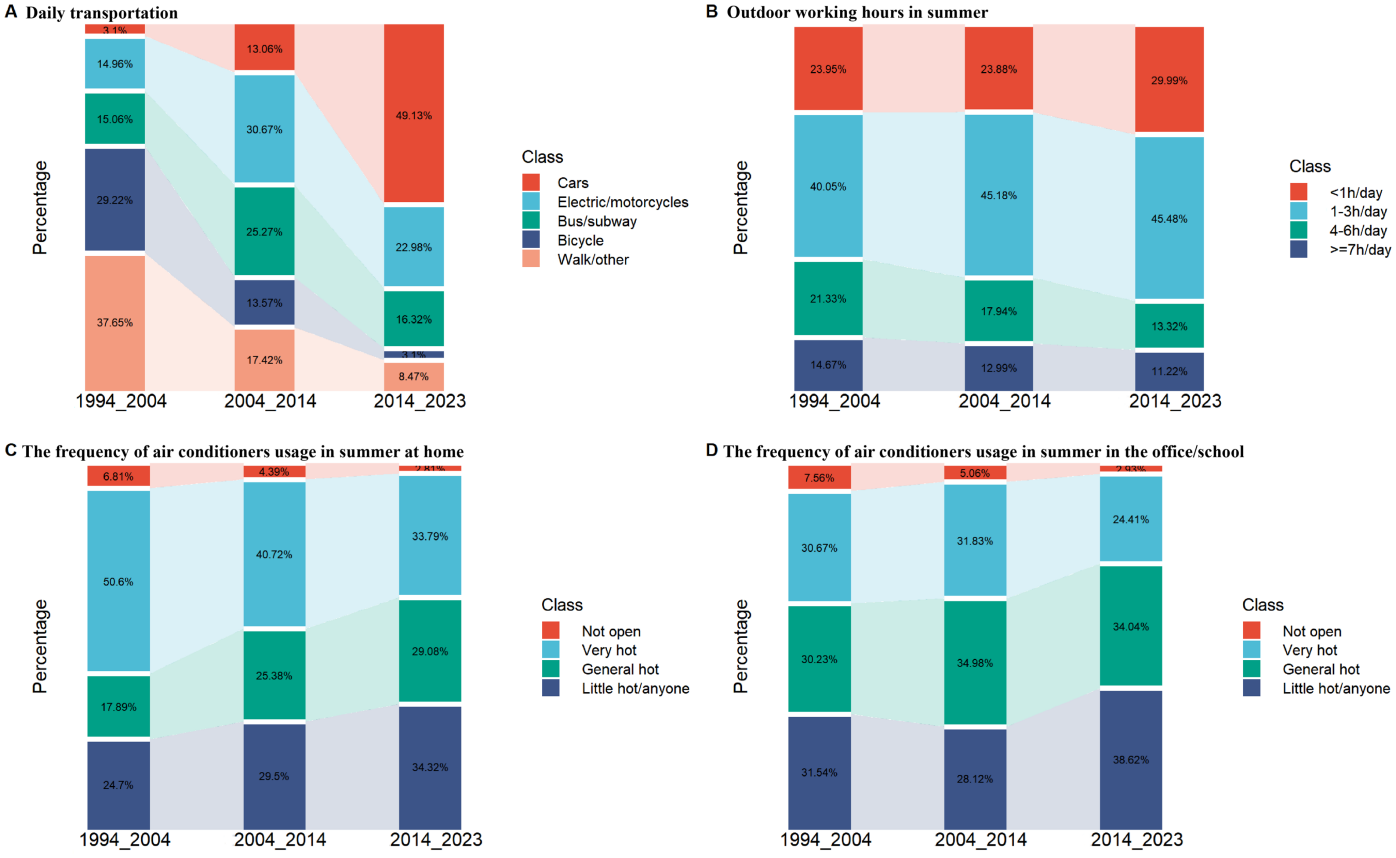
The temporal changes of heat exposure and temperature adaptation capacity during the last three decades from 1994 to 2023. Main daily transportation tools **(A)**; outdoor working hours in summer **(B)**; the frequency of air conditioners usage in summer at home **(C)** or in the office/school **(D)**.

Figure 2A presents the temporal changes of transportation tools in the last three decades. From 1994–2003, 2004–2013 to 2014–2023, private car significantly increased from 3.10, 13.06 to 49.13%, while bicycling (from 29.22, 13.57 to 3.10%) and waking (from 33.10, 14.32 to 6.56%) decreased significantly. For electric/motorcycles and buses/subways, they increased firstly and then decreased. The difference in transportation tools between the last three decades was statistically significant (CMH value = 156.40, *p* < 0.0001). Young people, females, outdoor workers, those with less education, low-income earners, and residents of southern China had higher waking/bicycling transportation tools usage (Table 2).

**Table 2:** The comparison of air conditioner ownership rate, AC usage frequency, the working time outdoors, and transportation tools during 2014–2023 between different demographic characteristics subgroups.

Subgroups	Air conditioner ownership rate				Air conditioners usage				Working time outdoors		Transportation tools			
	At home	*p value*	In the office/school	*p value*	At home	*p value*	In the office/school	*p value*	≧4 h	*p value*	Waking/ bicycling	Electric/motorcycles/buses/ subways	Private car	*p value*
Total	2,522(49.5)		2,491(50.3)											
Age(year)		<0.0001		<0.0001		<0.0001		<0.0001		0.095				<0.0001
31–40	1,309(84.7)		1,304(84.4)		449(35.1)		263(20.6)		399(25.8)		125(44.2)	672(44.2)	725(47.6)	
41–64	1,213(78.3)		1,187(76.6)		528(44.2)		354(30.4)		360(23.2)		174(36.0)	544(36.0)	795(52.5)	
Gender		0.076		0.166		0.033		0.207		<0.0001				<0.0001
Male	1,090(83.0)		1,073(81.7)		397(37.1)		252(24.0)		450(34.3)		110(36.9)	476(36.9)	705(54.6)	
Female	1,432(80.5)		1,418(79.7)		580(41.3)		365(26.2)		309(17.4)		189(42.4)	740(42.4)	815(46.7)	
Occupation types		<0.0001		<0.0001		0.017		0.003		<0.0001				0.001
Outdoor	124(61.4)		123(60.9)		45(38.1)		35(31.0)		164(81.2)		17(52.6)	103(52.6)	76(38.8)	
Half indoors and half out door	404(78.6)		395(76.9)		181(45.9)		120(31.3)		230(44.8)		57(35.4)	178(35.4)	268(53.3)	
Indoors	1994(83.9)		1973(83.0)		752(38.3)		462(23.8)		365(15.4)		225(40.0)	935(40.0)	1,176(50.3)	
Education		<0.0001		<0.0001		<0.0001		<0.0001		<0.0001				<0.0001
High school or below	314(68.7)		283(61.9)		149(49.3)		100(37.6)		241(52.7)		55(52.5)	231(52.5)	154(35.0)	
Undergraduate	1,742(81.9)		1,742(81.9)		692(40.4)		548(26.7)		465(21.9)		192(36.0)	752(36.0)	1,147(54.9)	
Graduate or above	466(91.2)		466(91.2)		136(29.7)		59(12.8)		53(10.4)		52(46.2)	233(46.2)	219(43.5)	
Income (yuan)		<0.0001		<0.0001		<0.0001		<0.0001		<0.0001				<0.0001
<5,000	782(76.0)		753(73.2)		408(53.1)		269(36.8)		283(27.5)		128(47.6)	477(47.6)	397(39.6)	
5,001–10,000	962(80.8)		960(80.6)		342(36.3)		217(23.2)		316(26.5)		103(37.8)	441(37.8)	623(53.4)	
>10,000	778(89.0)		788(89.02)		227(29.7)		131(16.9)		160(18.3)		68(34.4)	298(34.4)	500(57.7)	
Region		<0.0001		<0.0001		0.002		0.0009		<0.0001				<0.0001
Southern	2,134(89.4)		2,132(89.3)		806(38.3)		506(24.1)		512(21.4)		200(42.2)	992(42.2)	1,158(49.3)	
Northern	388(55.0)		358(50.7)		171(46.7)		111(32.6)		247(35.0)		99(32.7)	224(32.7)	362(52.9)	
Number of chronic diseases		<0.0001		<0.0001		0.488		0.572		<0.0001				0.197
0	1,644(83.3)		1,641(83.2)		651(40.1)		399(24.8)		425(21.5)		186(40.8)	789(40.8)	961(49.6)	
1	581(81.7)		567(79.8)		220(39.3)		140(25.4)		198(27.9)		68(41.3)	288(41.3)	341(48.9)	
≧2	297(72.4)		283(69.0)		106(36.4)		78(27.8)		136(33.2)		45(34.6)	139(34.6)	218(54.2)	

Figure 2B illustrates the changes in outdoor working hours over the last three decades. Between 1994–2003, 2004–2013, and 2014–2023, the proportion of individuals working outdoors for at least 4 h decreased significantly from 36.01 to 30.93%, and further to 24.53%. Conversely, the percentage of those working fewer than 4 h outdoors increased from 63.99 to 69.07%, and then to 75.47%. These differences were statistically significant (Z = −9.80, *p* < 0.001). Males, outdoor workers, those with less education, low-income earners, residents of northern China, and people with ≧2 chronic diseases had higher rates of outdoors working time ≧4 h (Table 2).

### The temporal change of adaptation capacity during the last three decades

Our analysis revealed that between the periods of 1994–2003, 2004–2013, and 2014–2023, there was a statistically significant increase in air conditioner ownership rates among the total population, from 25.40 to 81.51% at home (Z = −44.35, *p* < 0.0001) and from 22.24 to 80.51% in office/school settings (Z = −45.95, *p* < 0.0001) (Table 3). Stratified analyses by age, sex, workplace, education level, income, geographical region, and number of chronic diseases showed consistent patterns of temporal change. Table 2 presented subgroup analysis highlighting disparities during 2014–2023. Specifically, the older adult, outdoor workers, those with less education, low-income earners, and residents of northern China reported lower air conditioning ownership rates. Individuals with two or more chronic diseases (72.4%) and those with one chronic disease (81.7%) had lower ownership rates compared to those without chronic conditions (83.3%) (*p* < 0.001). The air conditioner ownership rates at home (83.0%) for male were not statistically different from that for female (80.5%) (*p* = 0.076). There was also no difference in the office (81.7% for male, 79.7% for female). However, after adjusting confounding factors, besides chronic health conditions, the older adult, females, outdoor workers, those with less education, low-income earners, and residents of northern China were independent predictors of non-ownership of air conditioners both domestically and in workplaces in multivariate logistic regression confirmed (Table 4).

**Table 3:** The difference comparison of air conditioner ownership rate at home and in the office/school between last three decades.

Subgroups	At home			Z value	*p* value	In the office/school			Z value	*p* value
	1994–2003	2004–2013	2014–2023			1994–2003	2004–2013	2014–2023		
	*N*(%)	*N*(%)	*N*(%)			*N*(%)	*N*(%)	*N*(%)		
Total	786(15.4)	1,783(35.0)	2,522(49.5)	−44.4	<0.0001	688(13.9)	1,778(35.9)	2,491(50.3)	−45.9	<0.0001
Age(year)										
31–40	288(18.6)	796(51.5)	1,309(84.7)	−36.8	<0.0001	235(15.2)	837(54.2)	1,304(84.4)	−38.5	<0.0001
41–64	498(32.2)	987(63.7)	1,213(78.3)	−26.0	<0.0001	453(29.2)	941(60.8)	1,187(76.6)	−26.5	<0.0001
Gender										
Male	314(23.9)	738(56.2)	1,090(83.0)	−30.4	<0.0001	305(23.2)	748(56.9)	1,073(81.7)	−30.1	<0.0001
Female	472(26.5)	1,045(58.7)	1,432(80.5)	−32.4	<0.0001	383(21.5)	1,030(57.9)	1,418(79.7)	−34.8	<0.0001
Occupation										
Outdoor	49(24.3)	75(37.1)	124(61.4)	−7.6	<0.0001	49(24.3)	72(35.6)	123(60.9)	−7.5	<0.0001
Half indoors and half out door	116(22.6)	271(52.7)	404(78.6)	−40.2	<0.0001	122(23.7)	260(50.6)	395(76.9)	−17.0	<0.0001
Indoors	621(26.1)	1,437(60.4)	1994(83.9)	−18.0	<0.0001	517(21.7)	1,446(60.8)	1973(83.0)	−42.5	<0.0001
Education										
High school or below	98(21.4)	195(42.7)	314(68.7)	−14.4	<0.0001	92(20.1)	170(37.2)	283(61.9)	−12.9	<0.0001
Undergraduate	537(25.3)	1,250(58.8)	1,742(81.9)	−37.2	<0.0001	499(23.5)	1,274(59.9)	1,742(81.9)	−38.3	<0.0001
Graduate or above	151(29.6)	338(66.2)	466(91.2)	−20.3	<0.0001	97(19.0)	334(65.4)	466(91.2)	−23.4	<0.0001
Income (yuan)										
<5,000	170(16.5)	479(46.6)	782(76.0)	−27.1	<0.0001	175(17.0)	470(45.7)	753(73.2)	−25.6	<0.0001
5,001–10,000	317(26.6)	694(58.3)	962(80.8)	−26.6	<0.0001	276(23.2)	687(57.7)	960(80.6)	−28.1	<0.0001
>10,000	299(34.2)	610(69.8)	778(89.0)	−23.9	<0.0001	237(27.1)	621(71.05)	788(89.02)	−26.7	<0.0001
Region										
Southern	660(27.7)	1,642(64.6)	2,134(89.4)	−43.6	<0.0001	564(23.6)	1,557(65.2)	2,132(89.3)	−46.2	<0.0001
Northern	125(17.7)	241(34.1)	388(55.0)	−14.6	<0.0001	123(17.4)	220(31.2)	358(50.7)	−13.2	<0.0001
Number of chronic diseases										
0	451(22.9)	1,137(57.6)	1,644(83.3)	−38.1	<0.0001	397(20.1)	1,161(58.8)	1,641(83.2)	−39.7	<0.0001
1	198(27.9)	419(58.9)	581(81.7)	−20.5	<0.0001	163(22.9)	417(58.7)	567(79.8)	−21.9	<0.0001
≧2	137(33.4)	227(55.4)	297(72.4)	−11.2	<0.0001	128(31.2)	200(48.8)	283(69.0)	−10.8	<0.0001

**Table 4:** Risk factors of low air conditioner ownership rate and low frequency of air conditioners usage at home or in the office/school based on multivariate logistic regression analysis.

Variable	Non-ownership of air conditioners				Low frequency of air conditioners usage until very hot			
	At home		In the office/school		At home		In the office/school	
	OR(95%CI)	*p* value	OR(95%CI)	*p* value	OR(95%CI)	*p* value	OR(95%CI)	*p* value
Age (year)		<0.0001		<0.0001		0.0005		<0.0001
31–40	1.00		1.00		1.00		1.00	
41–64	1.62(1.32–1.99)	<0.0001	1.75(1.41–2.15)	<0.0001	1.53(1.29–1.82)	<0.0001	1.64(1.36–1.99)	<0.0001
Gender							–	–
Male	1.00		1.00		1.00			
Female	1.51(1.21–1.88)	0.0002	1.46(1.17–1.81)	0.0008	1.21(1.01–1.44)	0.037		
Occupation types							–	–
Outdoor	1.00		1.00		1.00			
Half indoors and half out door	0.71(0.47–1.07)	0.880	0.98(0.64–1.48)	0.236	1.43(0.92–2.22)	0.024		
Indoors	0.48(0.33–0.71)	<0.0001	0.67(0.45–0.98)	0.003	1.05(0.7–1.58)	0.318		
Education					–	–		
High school or below	1.00		1.00				1.00	
Undergraduate	0.74(0.56–0.97)	0.246	0.47(0.36–0.62)	0.486			0.74(0.56–0.98)	0.186
Graduate or above	0.41(0.26–0.63)	0.0001	0.27(0.17–0.41)	<0.0001			0.4(0.27–0.6)	<0.0001
Income (yuan)								
<5,000	1.00		1.00		1.00		1.00	
5,001–10,000	0.49(0.37–0.66)	<0.0001	0.43(0.32–0.57)	<0.0001	0.37(0.3–0.46)	<0.0001	0.41(0.31–0.53)	<0.0001
>10,000	0.80(0.64–1.01)	0.220	0.69(0.55–0.87)	0.586	0.51(0.42–0.62)	0.032	0.56(0.45–0.7)	0.195
Region								
Southern	1.00		1.00		1.00		1.00	
Northern	7.14(5.8–8.79)	<0.0001	9.08(7.33–11.23)	<0.0001	1.67(1.32–2.12)	<0.0001	1.82(1.4–2.36)	<0.0001
Number of chronic diseases	–	–	–	–			–	–
0					1.00			
1					0.93(0.76–1.14)	0.463		
≧2					0.73(0.56–0.96)	0.046		

Figure 2C illustrates the changes in air conditioner usage over the past three decades. Between 1994–2003, 2004–2013, and 2014–2023, there was a significant increase in participants using air conditioners once they felt hot at home (from 17.89 to 25.38%, then to 29.08%), and when they felt slightly hot (from 24.70 to 29.50%, then to 34.32%). The differences were statistically significant (CMH value = 156.40, *p* < 0.0001). A similar pattern was observed in the office/school settings (CMH value = 65.29, *p* < 0.0001) (Figure 2D). The comparison between different demographic characteristics groups during 2014–2023 also emphasizes these difference in Table 2. The frequency of air conditioner usage was statistically different between at home for male (37.10%) and female (41.34%) (*p* = 0.033). However, the difference were not statistical between male (24.0%) and female (26.2%) in the office (*p* = 0.207). For chronic health conditions, there were no difference either at home or in the office (Table 2). In further multivariate logistic regression analysis during 2014–2023(Table 4), the older adult, females, outdoor workers, low-income earners, residents of northern China, and ≧2 chronic diseases were independent risk factors for low frequency of air conditioners usage at home (open until very hot). In terms of the office, the older adult, those with less education, low-income earners, residents of northern China were independent risk factors.

## Discussion

This study investigated the population’s knowledge and practice on climate change and health, and the temporal change of heat exposure and adaptation capacity during 1994–2023 based on an online survey. We found most people perceived increased temperature and changed behaviors to adapt to heat. During the last three decades, Chinese people reduced heat exposure by increasing the ownership rate of private car, reducing bicycling and working outdoors during hot season, and increased adaptation capacity by improving the ownership rate and usage of air conditioners at home and in the workplace. Our findings imply that people might adapt to global warming through behavioral and technological change when facing climate change.

Previous studies found knowledge and attitude of climate change may affect adaptation capacity and practice (27). In this study, we found most Chinese adults (94.6%) perceived increased temperature compared with their childhood, and over half (57.0%) people thought climate change impacted their health. This is consistent with previous studies (28, 29). For instance, a national survey in China found that 93.4% of respondents knew climate change (29). Another survey conducted in primary schools in 12 cities of China reported that 76.1% children were aware of climate change (28). This heterogeneity in different studies may be due to various demographic characteristics, geographic location and socioeconomic development level (29). For instance, our study did not include individuals aged 65 years and above, which may lead to the difference of our results with previous research, since a recent review found heat was not recognized as a threat by the older adult, and their risk perception of future heatwaves was also low (30, 31).

People can take various adaptation practices when feeling hot in summer. In our study, we observed that using air conditioners and fans, reducing outdoor activities, opening windows, and wearing thin clothes were top 5 adaptation practices. These findings were consistent with several previous studies (21, 30, 32–35). For instance, a review reported the diversity of adaptation behaviors, and clothing insulation, fan and air conditioner usage, and opening window were the most common adaptation practices (32). Another study conducted in China also found drinking more water, wearing light clothes and decreasing activities were major adaptation practices in hot weather (33). This finding implies that people would change behaviors to adapt to hot weather, which may significantly reduce heat-related adverse health impact.

The magnitude of health risk attributed to heat is closely related to exposure duration. Our study found that heat exposure including work and transportation exposure among Chinese adults significantly decreased in the past three decades. Transportation exposure reduction could be explained by economic development, since economy is highly associated with transportation tool change. Before the 1990s, bicycling and walking were the main transportation tools in China. With economic development, since the 1990s, motorcycles, buses and subways have gradually become popular in China. After the 2010s private cars were very popular in China and the importance of motorcycles and buses declined. In terms of outdoor working time, in the past three decades, due to economic development and technological advancements, China’s employment structure has undergone a significant transformation from being dominated by the primary and secondary industries to being dominated by the tertiary industry, which may significantly reduce heat exposure. This finding suggests that with socio-economic development, people may reduce heat exposure through changing transportation tools and reducing outdoor working time.

Our analysis further revealed a substantial rise in both the ownership rates and usage frequency of air conditioning systems both at home and in the office. This trend aligns with findings from prior research (32, 33, 36). For instance, a study in Shanghai of China reported the number of air conditioners per 100 households increased from almost 0 in 1981 to 96.4 in 2000, and 207 in 2012 (12).The proportion of households equipped with air conditioning escalated from 9.0% in 1993 to 19.2% in 2003 in Spain. Comparatively, Canada witnessed an increase from 30.1% in 1994 to 41.9% in 2003; Japan saw a rise from 73.7% in 1995 to 86.0% in 2003 (37). In the United States, the figures stood at 76.8% in 1994, progressing to 82.8% by 2004 (21). The increased air conditioner penetration might partly explain the decreased trend of heat-related health effect observed in previous studies (2, 9–11). However, higher penetration of air conditioner means higher energy demand, leading to greenhouse emission, which may exacerbate climate change (38). Therefore, developing green energy or more diverse adaptation measures are necessary in the future, such as developing heatwave early warning systems and emergency response systems, improving risk communication among vulnerable populations such as children, the older adult and pregnant woman, increasing public cooling areas and green space, using evaporative air cooler. In addition, the present study found that people more often used air conditioner in summer. Although there were few previous studies exploring the usage frequency of air conditioner, this result provided new evidence on increased adaptation capacity of heat among adults in China.

In this study, we further found the older adult, women, low income, outdoor work, low education, and people from northern China had lower rates of AC ownership. This finding was in line with previous studies (21, 34, 39, 40). For instance, a research in USA reported that the older adult and individuals with low incomes could not afford air conditioner (34). Even if they had air conditioner, they might not use it during extreme heat, since the electricity cost of air conditioners usage was expensive (41). This finding suggest that disadvantaged population had lower adaptation capacity of heat, which make them vulnerable population when exposure to extreme heat events in the context of climate change.

This study examined the long-term temporal change of heat exposure and adaptation capacity in China from 1994 to 2023, which provides valuable information for future adaptation. Besides, this study covered detailed demographic information on age, sex, income, occupation, education, income, region and number of chronic disease, offering depth on how different groups adapt. However, data in this study were self-reported, which might lead to reporting bias. Moreover, our study was based on an online survey rather than a randomized sample. Due to the limitation of convenience sampling design, our results were hard to extrapolate total population. Third, fewer middle-aged and old people were included in the study since they could not access internet, which might make temperature adaptation capacity be overestimated in our study. However, since all data in the past three decades did not include them, so our results might be comparative between the three decades. In the future, a multi-stage stratified randomized sampling rather than convenience sampling should be used to improve sample representation. Barriers or challenges people faced in adaptation capacity (e.g., cost of air conditioning, cultural preferences) will be further investigated in the future study.

## Conclusion

This study found that most people perceived climate change. Heat exposure significantly decreased and temperature adaptation capacity significantly increased. The findings implied that heat-related health risk and burden driven by global warming may not increase in the future due to continuously improved adaptation capacity.

## Data Availability

The raw data supporting the conclusions of this article will be made available by the authors, without undue reservation.
