# Supplementary material for: The temporal change of heat exposure and adaptation capacity in Chinese adults from 1994 to 2023
Source: Front Public Health. 2025 Jan 28;12:1492523. doi: 10.3389/fpubh.2024.1492523 (PMC11812521; doi:10.3389/fpubh.2024.1492523)
Supplement: Supplementary file 1 [file Table_1.doc]

Questionnaire on Adaptation capacity to High Temperature or Heatwaves

Dear survey subject:

Thank you for your participation. The purpose of the survey is to understand your temperature adaptation capacity and behaviors. This survey is conducted anonymously, and all data are confidential. Answer is no right or wrong for each question, please fill in the questionnaire according to your real situation.

Q1. Gender:① Male ② Female

Q2. Date of birth: _____Day_____Month_____Year

Q3.In the past 5 years during 2019 to 2023, your home address: __ District __City __Province

Q4. In the past 5 years, your main occupation:

①indoor work ②outdoor work ③indoor and outdoor work

Q5.In the past 5 years, month income per person of your family:

①<2000 yuan ②2000-5000 yuan ③5000-10000 yuan ④10000-20000 yuan

⑤≥20000 yuan

Q6.Are you suffering from the following chronic diseases? [Multiple Choice]

①No ②Hypertension ③Diabetes ④Hyperlipidemia ⑤Cardiovascular and cerebrovascular diseases ⑥Tumors ⑦Respiratory system diseases

⑧Digestive system diseases ⑨Kidney diseases ⑩Neuro psychiatric disorders

⑪Hearing problems ⑫Vision problems ⑬Others________________

Q7. Have you installed air conditioner at your home in different decades

| Decades | ①Yes | ②No |
| --- | --- | --- |
| 1994-2003 |  |  |
| 1994-2003 |  |  |
| 2014-2023 |  |  |

Q8.How often do you turn on your air conditioner at your home during summer?

| Decades | ①  Someone  at home | ②  a bit hot | ③  very hot | ④  Not open,  even very hot | ⑤No need to turn on, as the local weather is not hot | ⑥  No air conditioner |
| --- | --- | --- | --- | --- | --- | --- |
| 1994-2003 |  |  |  |  |  |  |
| 1994-2003 |  |  |  |  |  |  |
| 2014-2023 |  |  |  |  |  |  |

Q9.Did you installed air conditioner in your office/classroom during different decades

| Decades | ①Yes | ②No |
| --- | --- | --- |
| 1994-2003 |  |  |
| 1994-2003 |  |  |
| 2014-2023 |  |  |

Q10.How often did you turn on air conditioner in your office/school during summer?

| Decades | ①  Someone  at home | ②  a bit hot | ③  very hot | ④  Not open,  even very hot | ⑤No need to turn on, as the local weather is not hot | ⑥  No air condition |
| --- | --- | --- | --- | --- | --- | --- |
| 1994-2003 |  |  |  |  |  |  |
| 1994-2003 |  |  |  |  |  |  |
| 2014-2023 |  |  |  |  |  |  |

Q11.How many hours do you spend working outdoors within a day in summer in the following three decade?

| Decades | ① <1 hour | ② 1-4 hours | ③ 5-8 hours | ④ ≥8 hours |
| --- | --- | --- | --- | --- |
| 1994-2003 |  |  |  |  |
| 1994-2003 |  |  |  |  |
| 2014-2023 |  |  |  |  |

Q12.How often did you obtain heat information in the past 5 years?

①Almost every day ②5-6 days a week ③3-4 days a week

④1-2 days a week ⑤never

Q13.In the past 5 years, when you felt hot, which of the following measures did you mainly taken?[Multiple Choice]

①Turned on air conditioner ②Turned on fan

③Opened window for ventilation ④went to a place with air conditioning or shade ⑤Reduced outdoor activities ⑥Wore thin clothes or wore less clothes

⑦Used protective gear or sun umbrellas ⑧Used a handheld electric fan

⑨Took more showers ⑩Swam

⑪Drunk cold drinks, ate ice cream, watermelon, etc

⑫ Comfortable weather, I did not take any measures.

⑬Others________________

Q14. Which of the following statements about climate change is correct?

①Climate change is only caused by human activities.

②Climate change only refers to changes in temperature, excluding changes in dryness and wetness.

③Climate change includes changes in both mean temperature and extreme weather events.

④Global temperatures have been continuously decreasing over the past century.

Q15.Did you think the temperature in the past 5 years was lower than the temperature during your childhood?

①significantly increase ②increase ③no change ④decrease

⑤significantly decrease

Q16. In the past 5 years, how did you think high temperatures/heatwaves affected your health?

①most ②more ③ generally ④less ⑤least
